# Supplementary material for: Biodiversity measures of a grassland plant-pollinator community are resilient to the introduction of honey bees (Apis mellifera)
Source: PLoS One. 2024 Oct 25;19(10):e0309939. doi: 10.1371/journal.pone.0309939 (PMC11508496; doi:10.1371/journal.pone.0309939)
Supplement: S1 Table — The northernmost, central, and southernmost hive locations are listed as Bee48, Bee32, and Bee16 respectively, each associated number indicating the number of hives. Each transect is indicated by its treatment (100 m, 500 m, or 5000 m distance from a hive location). Letters indicate each replicate (See Fig 1). G5000 indicates the new position for F5000 that was moved mid-season. Reprinted from Worthy et al. [29] under a CC BY license, with permission from PLOS ONE, original copyright 2023. (DOCX) [file pone.0309939.s001.docx]

Table S1: Longitude and latitude for each hive location and transect, and collection effort at each transect. The northernmost, central, and southernmost hive locations are listed as Bee48, Bee32, and Bee16 respectively, each associated number indicating the number of hives. Each transect is indicated by its treatment (100 m, 500 m, or 5000 m distance from a hive location). Letters indicate each replicate (See Figure 1). G5000 indicates the new position for F5000 that was moved mid-season. Reprinted from Worthy et al [1] under a CC BY license, with permission from PLOS ONE, original copyright 2023.

| **Longitude** | **Latitude** | **Transect** | **Total Hand-caught Collections** | **Total Pan-trapped Collections** |
| --- | --- | --- | --- | --- |
| -111.91850 | 50.90210 | Bee48 |  |  |
| -111.94658 | 50.88120 | Bee32 |  |  |
| -111.93580 | 50.84320 | Bee16 |  |  |
| -111.91992 | 50.90202 | A100 | 10 | 10 |
| -111.92545 | 50.90248 | A500 | 10 | 10 |
| -111.98687 | 50.91594 | A5000 | 8 | 8 |
| -111.91699 | 50.90205 | B100 | 7 | 10 |
| -111.91010 | 50.90265 | B500 | 6 | 6 |
| -111.85447 | 50.88185 | B5000 | 8 | 8 |
| -111.94792 | 50.88178 | C100 | 9 | 9 |
| -111.95325 | 50.88365 | C500 | 8 | 8 |
| -112.00554 | 50.90691 | C5000 | 8 | 8 |
| -111.94533 | 50.88081 | D100 | 9 | 9 |
| -111.94149 | 50.87831 | D500 | 9 | 9 |
| -111.87483 | 50.86788 | D5000 | 8 | 6 |
| -111.93735 | 50.84338 | E100 | 3 | 10 |
| -111.94283 | 50.84407 | E500 | 8 | 10 |
| -111.99767 | 50.93795 | E5000 | 7 | 6 |
| -111.93415 | 50.84310 | F100 | 8 | 10 |
| -111.92928 | 50.84510 | F500 | 7 | 8 |
| -111.86687 | 50.83544 | F5000 | 2 | 2 |
| -111.98858 | 50.95827 | G5000 | 5 | 3 |

# References

1. Worthy SH, Acorn JH, Frost CM. Honey bees (Apis mellifera) modify plant-pollinator network structure, but do not alter wild species’ interactions. Mansour R, editor. PLoS ONE. 2023;18: e0287332. doi:10.1371/journal.pone.0287332
