# Supplementary material for: Biodiversity measures of a grassland plant-pollinator community are resilient to the introduction of honey bees (Apis mellifera)
Source: PLoS One. 2024 Oct 25;19(10):e0309939. doi: 10.1371/journal.pone.0309939 (PMC11508496; doi:10.1371/journal.pone.0309939)
Supplement: S2 Table — Full citations are listed in the References section of the Supporting Information. Reprinted from Worthy et al. [29] under a CC BY license, with permission from PLOS ONE, original copyright 2023. (DOCX) [file pone.0309939.s002.docx]

Table S2. List of references and resources used in species identifications. Full citations are listed in the References section of the Supporting Information. Reprinted from Worthy et al [1] under a CC BY license, with permission from PLOS ONE, original copyright 2023.

| **Insect Group** | **References** | **Museum Resources** | **Expert Taxonomists** |
| --- | --- | --- | --- |
| Butterflies  (Lepidoptera - Papilionoidea) | - Acorn (1993) - [2] - Vandyk (2021) - [3] - Warren et al (2016) - [4] | - University of Alberta Strickland Museum | John Acorn |
| Moths (Lepidoptera) |  | - Northern Forestry Centre | Greg Pohl |
| Ants (Hymenoptera – Formicoidea) | - Glasier et al (2013) - [5] |  | James Glasier |
| Beetles (Coleoptera) | - Acorn (2001) - [6] - Acorn (2007) - [7] - Arnett and Thomas (2000) - [8] - Arnett et al (2002) - [9] - Bousquet et al (2013) - [10] - Lindroth (1961) - [11] - Pinto (1991) - [12] - Smith and Skelley (2020) - [13] - Vandyk (2021) - [3] | - University of Alberta Strickland Museum | John Acorn |
| Flies (Diptera) | - Aldrich (1926) - [14] - Aldrich (1928) - [15] - Ávalos-Hernández (2009) - [16] - Brooks (1943a) - [17] - Brooks (1943b) - [18] - Brooks (1945) - [19] - Burt (2015) - [20] - Camras (1944) - [21] - Camras (1945) - [22] - Camras (1957) - [23] - Cole (1923) - [24] - Coquillett (1897) - [25] - Curran (1921) - [26] - Curran (1923) - [27] - Curran (1935) - [28] - Curran (1939) - [29] - Curran (1941) - [30] - Curran and Fluke (1926) - [31] - Fluke and Weems (1956) - [32] - Foster and Mathis (2012) - [33] - Gibson (2017) - [34] - Gill (1962) - [35] - Hardy (1943) - [36] - Huckett (1954) - [37] - James (1936) - [38] - James (1974) - [39] - James and Steyskal (1952) - [40] - Kits et al. (2008) - [41] - Malloch (1918) - [42] - Malloch (1920) - [43] - Malloch (1921) - [44] - Martin (1959) - [45] - McAlpine (1981) - [46] - McAlpine (1993) - [47] - Miranda et al (2013) - [48] - O’Hara (1982) - [49] - O’Hara and Wood (2004) - [50] - Sabrosky (1935) - [51] - Sabrosky (1955) - [52] - Sabrosky (1967) - [53] - Schlinger (1960) - [54] - Sedman (1966) - [55] - Shannon (1926) - [56] - Shannon (1939) - [57] - Skevington and Thompson (2012) - [58] - Strickland (1938) - [59] - Sun and Marshall (2003) - [60] - Telford (1970) - [61] - Thompson (1981) - [62] - Thompson et al (1990) - [63] - Townsend (1891) - [64] - Townsend (1908) - [65] - Vockeroth (1992) - [66] - Webb et al (2013) - [67] - Whitworth (2006) - [68] - Wilder (1979) - [69] - Young et al (2016) - [70] | - University of Alberta Strickland Museum | Brittany Wingert |
| Bees (Hymenoptera - Anthophila) | - Cockerell (1896) - [71] - Cockerell (1897) - [72] - Cockerell (1902) - [73] - Cockerell (1936) - [74] - Cockerell (1937a) - [75] - Cockerell (1937b) - [76] - Cockerell (1937c) - [77] - Cresson (1869) - [78] - Cresson (1878) - [79] - Droege et al (2010) - [80] - Droege et al (2021) - [81] - Dumesh and Sheffield (2012) - [82] - Dumesh and Sheffield (2014) - [83] - Gibbs (2010) - [84] - Hurd and Michener (1955) - [85] - LaBerge (1956) - [86] - LaBerge (1961) - [157] - McGinley (1986) - [88] - Michener (2007) - [89] - Mitchell (1956) - [90] - Mitchell (1960) - [91] - Mitchell (1962) - [92] - Mitchell (1973) - [93] - Onuferko (2017) - [94] - Packer et al (2007) - [95] - Rightmyer (2008) - [96] - Roberts (1973a) - [97] - Roberts (1973b) - [98] - Sheffield et al. (2011) - [99] - Sheffield et al. (2014) - [100] - Stephen (1954) - [101] - Viereck and Cockerell (1914) - [102] - Williams et al (2014) - [103] - York University (n.d.) - [104] | - University of Alberta Strickland Museum - University of Calgary Museum of Zoology | Lincoln Best |
| Wasps (Hymenoptera - Apocrita) | - Goulet and Huber (1993) - [105] - Kimsey and Carpenter (2012) - [106] | Royal Alberta Museum | Matthias Buck |
| Plants | - Bain et al (2014) - [107] - Moss (1994) - [108] - Tannas (2003) - [109] - Tannas (2004) - [110] |  |  |

# References

1. Worthy SH, Acorn JH, Frost CM. Honey bees (Apis mellifera) modify plant-pollinator network structure, but do not alter wild species’ interactions. Mansour R, editor. PLoS ONE. 2023;18: e0287332. doi:10.1371/journal.pone.0287332

2. Acorn J. Butterflies of Alberta. Edmonton: Lone Pine Publishing; 1993.

3. Vandyk J, editor. BugGuide.Net: Identification, Images, & Information For Insects, Spiders & Their Kin For the United States & Canada. Iowa State University; 2021. Available: https://bugguide.net/

4. Warren AD, Davis KJ, Strangeland EM, Pelham JP, Willmott KR, Grishin NV. Butterflies of America. In: Illustrated Lists of American Butterflies (North and South Amerca) [Internet]. 2016 [cited 29 Nov 2021]. Available: https://www.butterfliesofamerica.com/

5. Glasier JRN, Acorn, John H., Nielsen SE, Proctor H. Ants (Hymenoptera: Formicidae) of Alberta: A key to species based primarily on the worker caste. CJAI. 2013;22. doi:doi:10.3752/cjai.2013.22

6. Acorn J. The Tiger Beetles of Alberta: Killers on the Clay, Stalkers on the Sand. Edmonton: University of Alberta Press; 2001.

7. Acorn J. Ladybugs of Alberta: finding the spots and connecting the dots. 1st ed., 1st print., 2007. Edmonton: University of Alberta Press; 2007.

8. Arnett RH, Thomas MC, editors. American beetles, Volume I: Archostemata, Myxophaga, Adephaga, Polyphaga: Staphyliniformia. Boca Raton, Fla: CRC Press; 2000.

9. Arnett RH, Thomas MC, Skelley PE, Frank JH, editors. American beetles, Volume II: Polyphaga: Scarabaeoidea through Curculionoidea. Boca Raton, Fla: CRC Press; 2002.

10. Bousquet Y, Bouchard P, Davies A, Sikes D. Checklist of beetles (Coleoptera) of Canada and Alaska. Second edition. ZK. 2013;360: 1–44. doi:10.3897/zookeys.360.4742

11. Lindroth CH. The Ground-Beetles (Carabidae, Excluding Cicindelinae) of Canada and Alaska. Opuscula Entomologica. 1961;Supplementa XX, XXIV, XXIX, XXXIII, XXXIV, XXXV,. doi:10.2307/2412348

12. Pinto JD. The taxonomy of North American Epicauta (Coleoptera: Meloidae), with a revision of the nominate subgenus and a survey of courtship behavior. Berkeley: University of California Press; 1991.

13. Smith ABT, Skelley PE. A New Species of Flaviellus Gordon and Skelley, 2007 (Coleoptera: Scarabaeidae: Aphodiinae) from the Yukon, Canada. The Coleopterists Bulletin. 2020;74: 101. doi:10.1649/0010-065X-74.1.101

14. Aldrich JM. North American two-winged flies of the genus Cylindromyia Meigen (Ocyptera of authors). Proceedings of the United States National Museum. 1926;68: 1–27. doi:10.5479/si.00963801.68-2624.1

15. Aldrich JM. A revision of the American parasitic flies belonging to the genus Belvosia. Proceedings of the United States National Museum. 1928;73: 1–45. doi:10.5479/si.00963801.73-2729.1

16. Ávalos-Hernández O. A Review Of The North American Species Of Hemipenthes Loew, 1869 (Diptera: Bombyliidae). 2009 [cited 28 Jan 2022]. doi:10.5281/ZENODO.187152

17. Brooks AR. A REVIEW OF THE CANADIAN SPECIES OF ERNESTIA SENS. LAT. (TACHINIDAE, DIPTERA). Can Entomol. 1943;75: 66–78. doi:10.4039/Ent7566-4

18. Brooks AR. A REVIEW OF THE NORTH AMERICAN SPECIES OF GONIA sens. lat. (DIPTERA, TACHINIDAE). Can Entomol. 1943;75: 219–236. doi:10.4039/Ent75219-12

19. Brooks AR. A REVISION OF THE NORTH AMERICAN SPECIES OF THE RHODOGYNE COMPLEX (DIPTERA, LARVAEVORIDAE). Can Entomol. 1945;77: 218–230. doi:10.4039/Ent77218-12

20. Burt T. Taxonomic revision of four Nearctic Conopidae (Insecta: Diptera) genera (Dalmannia, Roberstonomyia, Stylogaster and Zodion) with notes on all other Nearctic genera. Carleton University. 2015. Available: https://curve.carleton.ca/system/files/etd/83a38375-1d49-4c32-8c2c-2b0f0b9301a1/etd_pdf/1c9eb60a06629211d3fe3d6e7f5ffdff/burt-taxonomicrevisionoffournearcticconopidaeinsecta.pdf

21. Camras S. Notes on the North American species of the Zodion fulvifrons group (Diptera: Conopidae). The Pan-Pacific Entomologist. 1944;20: 121–128.

22. Camras S. A Study of the Genus Occemyia in North America (Diptera: Conopidae)1. Annals of the Entomological Society of America. 1945;38: 216–222. doi:10.1093/aesa/38.2.216

23. Camras S. A Review of the New World Physocephala (Diptera: Conopidae). Annals of the Entomological Society of America. 1957;50: 213–218. doi:10.1093/aesa/50.3.213

24. Cole FR. A revision of the North American two-winged flies of the family Therevidae. Proceedings of the United States National Museum. 1923;62: 1–140. doi:10.5479/si.00963801.62-2450.1

25. Coquillett DW. Revision of the Tachinidae of America north of Mexico: a family of parasitic two-winged insects. Washington: Government Printing Office; 1897. doi:10.5962/bhl.title.87236

26. Curran CH. Revision of the Pipiza group of the family Syrphidae (flower-flies) from north of Mexico. Proceedings of the California Academy of Sciences. 1921;11: 345–393.

27. Curran CH. Notes on the Genus Pipizella Rondani, with Descriptions of New Species (Diptera; Syrphidae). Transactions of the American Entomological Society. 1923;49: 339–345.

28. Curran CH. The families and genera of North American Diptera. American Museum Novitates. 1935;812. doi:10.5962/bhl.title.6825

29. Curran CH. The species of Temnostoma related to bombylans Linné (Syrphidae, Diptera). American Museum Novitates. 1939;1040.

30. Curran CH. New American Syrphidae. Bulletin of the American Museum of Natural History. 1941;78: 243–304.

31. Curran CH, Fluke CL. Revision of the nearctic species of Helophilus and allied genera. Transactions of the Wisconsin Academy of Sciences, Arts and Letters. 1926; 207–281.

32. Fluke CL, Weems JrHV. The Myoleptini of the Americas (Diptera, Syrphidae). American Museum Novitates. 1956;1758: 1–23.

33. Foster GA, Mathis WN. A revision of the nearctic species of the genus Trixoscelis Rondani (Diptera: Heleomyzidae: Trixoscelidinae). Smithsonian Contributions to Zoology. 2012; 1–128. doi:10.5479/si.00810282.637.1

34. Gibson JF. An updated and annotated checklist of the thick-headed flies (Diptera: Conopidae) of British Columbia, the Yukon, and Alaska. Journal of the Entomological Society of British Columbia. 2017;114.

35. Gill GD. The Heleomyzid Flies of American North of Mexico (Diptera: Heleomyzidae). Proceedings of the United States National Museum. 1962;113: 495–603. doi:10.5479/si.00963801.113-3465.495

36. Hardy E. New Nearctic Pipunculidae (Diptera). Journal of the Kansas Entomological Society. 1943;12: 16–25.

37. Huckett HC. A Review of the North American Species Belonging to the Genus Hydrotaea Robineau-Desvoidy (Diptera, Muscidae). Annals of the Entomological Society of America. 1954;47: 316–342. doi:10.1093/aesa/47.2.316

38. James MT. The Genus Odontomyia in America North of Mexico (Diptera, Stratiomyidae). Annals of the Entomological Society of America. 1936;29: 517–550. doi:10.1093/aesa/29.3.517

39. James MT. The Status of Odontomyia arcuata Loew, O. inaequalis Loew, and Their Close Relatives in Western North America (Diptera: Stratiomyidae). Journal of the Kansas Entomological Society. 1974;47: 222–226.

40. James MT, Steyskal GC. A Review of the Nearctic Stratiomyini (Diptera, Stratiomyidae). Annals of the Entomological Society of America. 1952;45: 385–412. doi:10.1093/aesa/45.3.385

41. Kits JH, Marshall SA, Evenhuis NL. The Bee Flies (Diptera: Bombyliidae) of Ontario, with a Key to the Species of Eastern Canada. CJAI. 2008;6.

42. Malloch JR. Diptera from the South-Western United States. Paper IV. Anthomyiidae. Transactions of the American Entomological Society. 1918;44: 263–319.

43. Malloch JR. A SYNOPTIC REVISION OF THE ANTHOMYIID GENUS HYDROPHORIA ROBINEAU-DESVOIDY (DIPTERA). Can Entomol. 1920;52: 253–257. doi:10.4039/Ent52253-11

44. Malloch JR. A SYNOPSIS OF THE NORTH AMERICAN SPECIES OF THE GENUS HELINA R.-D., SENS. LAT. (DIPTERA, ANTHOMYIIDAE). Can Entomol. 1921;53: 103–109. doi:10.4039/Ent53103-5

45. Martin CH. The Holopogon complex of North America, excluding Mexico, with the descriptions of a new genus and a new subgenus (Diptera, Asilidae). American Museum Novitates. 1959;1980.

46. McAlpine JF, editor. Manual of Nearctic Diptera. Vol. 1. Ottawa: Research Branch, Agriculture Canada; 1981.

47. McAlpine JF, editor. Manual of Nearctic Diptera. Vol. 2. Repr. Hull, Que: Canadian Government Publ. Centre; 1993.

48. Miranda GFG, Young AD, Locke MM, Marshall SA, Skevington JH, Thompson FC. Key to the Genera of Nearctic Syrphidae. CJAI. 2013;23: 1–351. doi:10.3752/cjai.2013.23

49. O’Hara JE. Classification, phylogeny and zoogeography of the North American species of Siphona Meigen (Diptera: Tachinidae). Department of Entomology, University of Alberta. 1982;18: 261–380.

50. O’Hara JE, Wood DM. Checklist of the Tachinidae (Diptera) of America north of Mexico. Online. 2004 [cited 28 Jan 2021]. Available: http://www.nadsdiptera.org/Tach/WorldTachs/TTimes/Tach18.html

51. Sabrosky CW. The Chloropidae of Kansas (Diptera). Transactions of the American Entomological Society. 1935;61: 207–268.

52. Sabrosky CW. A Third Species of Eusiphona, with Remarks on the Systematic Position of the Genus (Diptera, Milichiidae). Entomological News. 1955;66: 169–173.

53. Sabrosky CW. Notes on the tachinid genus Cylindromyia in North America. Proceedings of the Entomological Society of Washington. 1967;69: 60–63.

54. Schlinger EI. A Revision of the Genus Ogcodes Latreille with Particular Reference to Species of the Western Hemisphere. Proceedings of the United States National Museum. 1960;111: 227–336. doi:10.5479/si.00963801.111-3429.227

55. Sedman YS. The Chrysogaster (Orthonevra) pictipennis group in North America (Diptera: Syrphidae). Entomological Society of Washington. 1966;68: 185–194.

56. Shannon RC. Review of the American xylotine syrphid-flies. Proceedings of the United States National Museum. 1926;69: 1–52. doi:10.5479/si.00963801.2635

57. Shannon RC. Temnostoma bombylans and related species (Syrphidae, Diptera). Entomological Society of Washington. 1939;41: 215–224.

58. Skevington JH, Thompson FC. Review of New World Sericomyia (Diptera: Syrphidae), including description of a new species. Can Entomol. 2012;144: 216–247. doi:10.4039/tce.2012.24

59. Strickland EH. AN ANNOTATED LIST OF THE DIPTERA (FLIES) OF ALBERTA. Can J Res. 1938;16d: 175–219. doi:10.1139/cjr38d-012

60. Sun X, Marshall SA. Systematics of Phasia Latreille (Diptera: Tachinidae). Zootaxa. 2003;276: 1. doi:10.11646/zootaxa.276.1.1

61. Telford HS. Eristalis (Diptera: Syrphidae) from America North of Mexico1. Annals of the Entomological Society of America. 1970;63: 1201–1210. doi:10.1093/aesa/63.5.1201

62. Thompson FC. Revisionary notes on Nearctic Microdon flies (Diptera: Syrphidae). Proceedings of the Entomological Society of Washington. 1981;83: 725–758.

63. Thompson FC, Fee FD, Bezark LD. Two Immigrant Synanthropic Flower Flies (Diptera: Syrphidae) New to North America. Entomological News. 1990;101: 69–74.

64. Townsend CHT. NOTES ON NORTH AMERICAN TACHINIDÆ, WITH DESCRIPTIONS OF NEW GENERA AND SPECIES. Paper II. Can Entomol. 1891;18: 349–382.

65. Townsend CHT. The taxonomy of the muscoidean flies, including descriptions of new genera and species. Smithsonian Miscellaneous Collections. 1908;51: 1–138.

66. Vockeroth JR. The Flower Flies of the Subfamily Syrphinae of Canada, Alaska, and Greenland: Diptera: Syrphidae. Ottawa, Ont: Centre for Land and Biological Resources Research, Research Branch, Agriculture Canada; 1992.

67. Webb DW, Gaimari SD, Hauser M, Holston KC, Metz MA, Irwin ME, et al. An annotated catalogue of the New World Therevidae (Insecta: Diptera: Asiloidea). Zootaxa. 2013;3600: 1–105. doi:10.11646/zootaxa.3600.1.1

68. Whitworth T. Keys to the Genera and Species of Blow Flies (Diptera: Calliphoridae) of America, North of Mexico. Proceedings of the Entomological Society of Washington. 2006;108: 689–725.

69. Wilder DD. Systematics of the Nearctic Ptilodexia Brauer and Bergenstamm (Diptera, Tachinidae). Proceedings of the California Academy of Sciences. 1979;42: 55.

70. Young AD, Marshall SA, Skevington JH. Revision of Platycheirus Lepeletier and Serville (Diptera: Syrphidae) in the Nearctic north of Mexico. Zootaxa. 2016;4082: 1. doi:10.11646/zootaxa.4082.1.1

71. Cockerell TDA. The Bees of the Genus Perdita F. Smith. Proceedings of the Academy of Natural Sciences of Philadelphia. 1896;48: 25–107.

72. Cockerell TDA. New species of Andrena from North America. The Entomologist. 1897;30: 305–309.

73. Cockerell TDA. North American bees of the Genus Andrena. Annals and Magazine of Natural History. 1902;9: 101–106. doi:10.1080/00222930208678552

74. Cockerell TDA. THE BEES OF ALBERTA.—I. Can Entomol. 1936;68: 274–277. doi:10.4039/Ent68274-12

75. Cockerell TDA. Bees of the Genus Sphecodes from Saskatchewan. The American Museum of Natural History; 1937.

76. Cockerell TDA. THE BEES OF ALBERTA—IV. Can Entomol. 1937;69: 113–114. doi:10.4039/Ent69113-5

77. Cockerell TDA. BEES OF ALBERTA. V. Can Entomol. 1937;69: 126–127. doi:10.4039/Ent69126-6

78. Cresson ET. A list of the North American species of the genus Anthophora, with descriptions of new species. Transactions of the American Entomological Society; 1869. pp. 289–293.

79. Cresson ET. Descriptions of new species of North American bees. Proceedings of the Academy of Natural Sciences of Philadelphia; 1878. pp. 181–221.

80. Droege S, Rightmyer MG, Sheffield CS, Brady SG. New synonymies in the bee genus Nomada from North America (Hymenoptera: Apidae). Zootaxa. 2010;2661: 1. doi:10.11646/zootaxa.2661.1.1

81. Droege S, Jean R, Orr M. Bee Genera of Eastern North America. In: Apoidea - Discover Life [Internet]. 2021 [cited 2 Aug 2020]. Available: https://www.discoverlife.org/20/q?search=Apoidea

82. Dumesh S, Sheffield CS. Bees of the Genus Dufourea Lepeletier (Hymenoptera: Halictidae: Rophitinae) of Canada. CJAI. 2012;18.

83. Dumesh S, Sheffield CS. Photographic keys to the bees of the Northwest Territories, Canada. Department of Environment and Natural Resources, Government of the Northwest Territories.; 2014.

84. Gibbs J. Revision of the metallic species of Lasioglossum (Dialictus) in Canada (Hymenoptera, Halictidae, Halictini). Zootaxa. 2010;2591: 1. doi:10.11646/zootaxa.2591.1.1

85. Hurd PD, Michener CD. The Megachiline bees of California (Hymenoptera : Megachilidae). University of California Press Berkeley and Los Angeles; 1955.

86. LaBerge WE. A Revision of the Bees of the Genus Melissodes in North and Central America. Parts I., II. (Hymenoptera, Apidae). The University of Kansas science bulletin. 1956;37: 911–1194. doi:10.5962/bhl.part.24549

87. LaBerge WE. A Revision of the Bees of the Genus Melissodes in North and Central America. Part III (Hymenoptera, Apidae). The University of Kansas science bulletin. 1961;42: 283–663. doi:10.5962/bhl.part.9821

88. McGinley RJ. Studies of Halictinae (Apoidea: Halictidae), I: Revision of New World Lasioglossum Curtis. Smithsonian Contributions to Zoology. 1986; 1–294. doi:10.5479/si.00810282.429

89. Michener CD. The Bees of the World. 2nd ed. Baltimore: Johns Hopkins University Press; 2007.

90. Mitchell TB. New Species of Sphecodes from the Eastern United States. Journal of the Elisha Mitchell Scientific Society. 1956;72: 206–222.

91. Mitchell TB. Bees of the Eastern United States, V1. Raleigh: North Carolina Agricultural Experiment Station Technical Bulletin; 1960.

92. Mitchell TB. Bees of the Eastern United States, V2. Raleigh: North Carolina Agricultural Experiment Station Technical Bulletin; 1962.

93. Mitchell TB. A subgeneric revision of the bees of the genus Coelioxys of the Western Hemisphere (Hymenoptera: Megachilidae). Department of Entomology, North Carolina State University; 1973.

94. Onuferko TM. Cleptoparasitic bees of the genus Epeolus Latreille (Hymenoptera: Apidae) in Canada. CJAI. 2017;30: 1–62.

95. Packer L, Genaro JA, Sheffield CS. The Bee Genera of Eastern Canada. CJAI. 2007;3: 1–32.

96. Rightmyer MG. A review of the cleptoparasitic bee genus Triepeolus (Hymenoptera: Apidae).-Part I. Zootaxa. 2008;1710: 1. doi:10.11646/zootaxa.1710.1.1

97. Roberts RB. Bees of northwestern America: Agapostemon (Hymenoptera: Halictidae). Technical Bulletin of the Agricultural Experiment Station. 1973;125: 1–23.

98. Roberts RB. Bees of northwestern America: Halictus (Hymenoptera: Halictidae). Technical Bulletin of the Agricultural Experiment Station. 1973;126. Available: https://static1.squarespace.com/static/5a849d4c8dd041c9c07a8e4c/t/5a9727410d9297d03d5f566c/1519855427669/Roberts+1973+Bees+of+Northwestern+America+Halictus.pdf

99. Sheffield CS, Ratti C, Packer L, Griswold T. Leafcutter and Mason Bees of the Genus Megachile Latreille (Hymenoptera: Megachilidae) in Canada and Alaska. CJAI. 2011;18. doi:doi: 10.3752/cjai.2011.18

100. Sheffield CS, Frier SD, Dumesh, S. The Bees (Hymenoptera: Apoidea, Apiformes) of the Prairies Ecozone with Comparisons to other Grasslands of Canada. 2014; 427–467.

101. Stephen WP. A Revision of the Bee Genus Colletes in America North of Mexico (Hymenoptera Colletidae). The University of Kansas Science Bulletin. 1954;36: 149–527.

102. Viereck HL, Cockerell TDA. New North American bees of the genus Andrena. Proceedings of the United States National Museum. 1914;48: 1–58. doi:10.5479/si.00963801.48-2064.1

103. Williams P, Thorp RW, Richardson L, Colla S. Bumble bees of North America: an identification guide. Princeton: Princeton University Press; 2014.

104. York University. Key to the Genera of Andreninae. York University; Available: https://www.yorku.ca/bugsrus/resources/keys/Andreninae/Images/Andreninae_A_Start/Andreninae_A_Start_Export.htm

105. Goulet H, Huber JT, editors. Hymenoptera of the world: an identification guide to families. Ottawa, Ontario: Centre for Land and Biological Resources Research; 1993.

106. Kimsey L, Carpenter J. The Vespinae of North America (Vespidae, Hymenoptera). JHR. 2012;28: 37–65. doi:10.3897/jhr.28.3514

107. Bain J, Flanagan J, Kuigt J. Common Coulee Plants of Southern Alberta. 2nd ed. University of Lethbridge Herbarium.; 2014.

108. Moss EH, Packer JG. Flora of Alberta. 2nd ed. University of Toronto Press; 1994. Available: http://www.jstor.org/stable/10.3138/j.ctt1287xvf

109. Tannas K. Common plants of the western rangelands, volume 2: Trees and Shrubs. Olds College; 2003.

110. Tannas K. Common plants of the western rangelands, volume 3: Forbs. Olds College; 2004.
