# Supplementary material for: Biodiversity measures of a grassland plant-pollinator community are resilient to the introduction of honey bees (Apis mellifera)
Source: PLoS One. 2024 Oct 25;19(10):e0309939. doi: 10.1371/journal.pone.0309939 (PMC11508496; doi:10.1371/journal.pone.0309939)
Supplement: S3 Table — Datasets 1 to 5 are derived from the hand-caught dataset, and datasets 6 to 10 are derived from the pan-trapped dataset. (DOCX) [file pone.0309939.s003.docx]

Table S3: Number of individual insect pollinators, excluding honey bees, that were caught per transect for datasets 1 to 10 (see Fig 2). Datasets 1 to 5 are derived from the hand-caught dataset, and datasets 6 to 10 are derived from the pan-trapped dataset.

| **Transect** | **Hand-caught datasets** | | | | | **Pan-trapped datasets** | | | | |
| --- | --- | --- | --- | --- | --- | --- | --- | --- | --- | --- |
|  | Full-season All-taxa  (1) | Full-season Bees  (2) | Full-season Flies  (3) | Mid-season All-taxa  (4) | Mid-season Bees  (5) | Full-season Bees  (6) | Full-season Beetles  (7) | Full-season Butterflies  (8) | Mid-season Bees  (9) | Mid-season Beetles  (10) |
| A100 | 110 | 59 | 35 | 34 | 15 | 443 | 63 | 146 | 145 | 36 |
| A500 | 120 | 81 | 17 | 60 | 44 | 635 | 136 | 100 | 230 | 91 |
| A5000 | 101 | 50 | 11 | 46 | 29 | 462 | 161 | 206 | 207 | 62 |
| B100 | 48 | 14 | 9 | 20 | 9 | 420 | 82 | 158 | 156 | 63 |
| B500 | 103 | 27 | 35 | 25 | 0 | 255 | 115 | 107 | 26 | 47 |
| B5000 | 76 | 26 | 41 | 18 | 12 | 274 | 58 | 57 | 173 | 43 |
| C100 | 110 | 44 | 46 | 31 | 24 | 418 | 69 | 55 | 73 | 34 |
| C500 | 125 | 22 | 75 | 46 | 10 | 278 | 87 | 153 | 134 | 48 |
| C5000 | 119 | 22 | 60 | 51 | 10 | 210 | 267 | 124 | 93 | 123 |
| D100 | 57 | 22 | 17 | 13 | 2 | 358 | 41 | 98 | 78 | 19 |
| D500 | 91 | 37 | 34 | 9 | 6 | 449 | 54 | 134 | 109 | 27 |
| D5000 | 53 | 28 | 13 | 15 | 12 | 383 | 200 | 188 | 100 | 21 |
| E100 | 15 | 4 | 3 | NA | NA | 466 | 153 | 166 | 252 | 95 |
| E500 | 124 | 49 | 42 | 59 | 27 | 504 | 215 | 125 | 199 | 130 |
| E5000 | 74 | 26 | 17 | 30 | 20 | 155 | 68 | 254 | 69 | 29 |
| F100 | 74 | 33 | 14 | 35 | 17 | 326 | 188 | 114 | 131 | 116 |
| F500 | 55 | 7 | 14 | 21 | 5 | 299 | 293 | 127 | 116 | 228 |
| F5000 | 25 | 21 | 4 | 23 | 19 | 103 | 10 | 6 | NA | NA |
| G5000 | 49 | 15 | 7 | 21 | 13 | 78 | 18 | 196 | 42 | 3 |
