# Supplementary material for: Biodiversity measures of a grassland plant-pollinator community are resilient to the introduction of honey bees (Apis mellifera)
Source: PLoS One. 2024 Oct 25;19(10):e0309939. doi: 10.1371/journal.pone.0309939 (PMC11508496; doi:10.1371/journal.pone.0309939)
Supplement: S4 Table — (DOCX) [file pone.0309939.s004.docx]

**Table S4:** Variance inflation factors calculated for both the full-season and mid-season to assess multicollinearity between variables.

| **Full Season** | |
| --- | --- |
| **Predictor variable** | **VIF** |
| Honey bee abundance | 3.59 |
| Flower species richness | 1.96 |
| Flower abundance | 3.51 |
| Collection effort | 1.41 |
| **Midseason** | |
| **Predictor variable** | **VIF** |
| Honey bee abundance | 5.54 |
| Flower species richness | 1.82 |
| Flower abundance | 5.48 |
| Collection effort | 1.24 |
