# Supplementary material for: Biodiversity measures of a grassland plant-pollinator community are resilient to the introduction of honey bees (Apis mellifera)
Source: PLoS One. 2024 Oct 25;19(10):e0309939. doi: 10.1371/journal.pone.0309939 (PMC11508496; doi:10.1371/journal.pone.0309939)
Supplement: S5 Table — Each metric was used as the response variable in a statistical test of the effect of honey bee abundance on that variable. Analyses for some higher taxa from the mid-season all-taxa dataset could not be completed when abundances were too low (flies, butterflies, ants, moths, true bugs). Interaction richness and interaction diversity were only analyzed for the full-season all-taxa dataset. (DOCX) [file pone.0309939.s005.docx]

**Table S5:** List of datasets and metrics calculated for each dataset (See Fig 2). Each metric was used as the response variable in a statistical test of the effect of honey bee abundance on that variable. Analyses for some higher taxa from the mid-season all-taxa dataset could not be completed when abundances were too low (flies, butterflies, ants, moths, true bugs). Interaction richness and interaction diversity were only analyzed for the full-season all-taxa dataset.

| **Datasets** | **Response Variables Tested** |
| --- | --- |
| **Hand-caught dataset** | |
| **1) Full-season all-taxa dataset** | Species richness |
|  | Species diversity |
|  | Abundance |
|  | Species composition |
|  | Interaction richness |
|  | Interaction diversity |
| **2) Full-season Non-*Apis* bees** | Non-*Apis* bee species richness |
|  | Non-*Apis* bee diversity |
|  | Non-*Apis* bee abundance |
|  | Non-*Apis* bee species composition |
| **3) Full-season Flies** | Fly species richness |
|  | Fly species diversity |
|  | Fly abundance |
|  | Fly species composition |
| **4) Mid-season all-taxa dataset** | Species richness |
|  | Species diversity |
|  | Abundance |
|  | Species composition |
| **5) Mid-season Non-*Apis* dataset** | Non-*Apis* bee species richness |
|  | Non-*Apis* bee species diversity |
|  | Non-*Apis* bee abundance |
|  | Non-*Apis* bee species composition |
| **Pan-trapped dataset** | |
| **6) Full-season Non-*Apis* dataset** | Non-*Apis* bee species richness |
|  | Non-*Apis* bee species diversity |
|  | Non-*Apis* bee abundance |
|  | Non-*Apis* bee species composition |
| **7) Full-season Beetles dataset** | Beetle species richness |
|  | Beetle species diversity |
|  | Beetle abundance |
|  | Beetle species composition |
| **8) Full-season Butterflies dataset** | Butterfly species richness |
|  | Butterfly species diversity |
|  | Butterfly abundance |
|  | Butterfly species composition |
| **9) Mid-season Non-*Apis* dataset** | Non-*Apis* bee species richness |
|  | Non-*Apis* bee species diversity |
|  | Non-*Apis* bee abundance |
|  | Non-*Apis* bee species composition |
| **10) Mid-season Beetles dataset** | Beetle species richness |
|  | Beetle species diversity |
|  | Beetle abundance |
|  | Beetle species composition |
