# Supplementary material for: Biodiversity measures of a grassland plant-pollinator community are resilient to the introduction of honey bees (Apis mellifera)
Source: PLoS One. 2024 Oct 25;19(10):e0309939. doi: 10.1371/journal.pone.0309939 (PMC11508496; doi:10.1371/journal.pone.0309939)
Supplement: S8 Table — Morphospecies identifications are listed by “[Genus] sp. #”. Some species could not be differentiated between genera, and so both genera are listed along with the epithet “sp”. Specimens listed beside “cf” (confer, meaning compare with) are specimens that were damaged or for which taxonomic keys are insufficient, and these were compared to other specimens to determine identification. Numbers of each (morpho)species are given for each distance from hives, despite the fact that we used honey bee abundance, rather than distance from hive as the predictor variable in our analyses. (DOCX) [file pone.0309939.s008.docx]

Table S8: Identifications of insect pollinators to species-level or morphospecies level from the pan-trapped dataset. Morphospecies identifications are listed by “[Genus] sp. #”. Some species could not be differentiated between genera, and so both genera are listed along with the epithet “sp”. Specimens listed beside “cf” (confer, meaning compare with) are specimens that were damaged or for which taxonomic keys are insufficient, and these were compared to other specimens to determine identification. Numbers of each (morpho)species are given for each distance from hives, despite the fact that we used honey bee abundance, rather than distance from hive as the predictor variable in our analyses.

| **Order/Group** | **Family** | **Species** | **100 m** | | **500 m** | **5000 m** | | **Total** |
| --- | --- | --- | --- | --- | --- | --- | --- | --- |
| **Anthophila** | | | | | | | | |
|  | Andrenidae | *Andrena amphibola* | 12 | 15 | | 9 | 36 | |
|  | Andrenidae | *Andrena barbilabris* | 7 | 14 | | 9 | 30 | |
|  | Andrenidae | *Andrena canadensis* | 0 | 0 | | 2 | 2 | |
|  | Andrenidae | *Andrena chromotricha* | 16 | 7 | | 8 | 31 | |
|  | Andrenidae | *Andrena lupinorum* | 4 | 8 | | 12 | 24 | |
|  | Andrenidae | *Andrena* cf. *medionitens* | 1 | 1 | | 2 | 4 | |
|  | Andrenidae | *Andrena* cf*. nigrihirta* | 0 | 0 | | 2 | 2 | |
|  | Andrenidae | *Andrena peckhami* | 10 | 9 | | 10 | 29 | |
|  | Andrenidae | *Andrena prunorum* | 6 | 4 | | 0 | 10 | |
|  | Andrenidae | *Andrena* cf. *sigmundi* | 2 | 1 | | 0 | 3 | |
|  | Andrenidae | *Andrena* sp. | 0 | 0 | | 1 | 1 | |
|  | Andrenidae | *Andrena* sp.1 | 0 | 1 | | 2 | 3 | |
|  | Andrenidae | *Andrena* sp.2 | 0 | 1 | | 0 | 1 | |
|  | Andrenidae | *Andrena* sp.3 | 0 | 1 | | 3 | 4 | |
|  | Andrenidae | *Andrena* sp.4 | 3 | 1 | | 4 | 8 | |
|  | Andrenidae | *Andrena* sp.5 | 0 | 0 | | 3 | 3 | |
|  | Andrenidae | *Andrena* sp.6 | 1 | 0 | | 0 | 1 | |
|  | Andrenidae | *Andrena* sp.7 | 0 | 1 | | 0 | 1 | |
|  | Andrenidae | *Andrena* sp.8 | 0 | 1 | | 0 | 1 | |
|  | Andrenidae | *Andrena* sp.9 | 1 | 2 | | 1 | 4 | |
|  | Andrenidae | *Andrena thaspii* | 7 | 5 | | 8 | 20 | |
|  | Andrenidae | *Andrena trevoris* | 1 | 0 | | 0 | 1 | |
|  | Andrenidae | *Panurginus beardsleyi* | 1 | 1 | | 0 | 2 | |
|  | Andrenidae | *Perdita bruneri* | 22 | 35 | | 9 | 66 | |
|  | Andrenidae | *Perdita* sp.1 | 1 | 0 | | 0 | 1 | |
|  | Andrenidae | *Perdita* sp.2 | 38 | 44 | | 15 | 97 | |
|  | Andrenidae | *Perdita swenki* | 3 | 2 | | 1 | 6 | |
|  | Apidae | *Anthophora bombiodes* | 1 | 0 | | 1 | 2 | |
|  | Apidae | *Anthophora occidentalis* | 3 | 7 | | 0 | 10 | |
|  | Apidae | *Anthophora porterae* | 3 | 0 | | 0 | 3 | |
|  | Apidae | *Anthophora terminalis* | 1 | 3 | | 1 | 5 | |
|  | Apidae | *Apis mellifera* | 103 | 53 | | 13 | 169 | |
|  | Apidae | *Bombus borealis* | 26 | 32 | | 34 | 92 | |
|  | Apidae | *Bombus fervidus* | 4 | 7 | | 14 | 25 | |
|  | Apidae | *Bombus griseocollis* | 0 | 0 | | 2 | 2 | |
|  | Apidae | *Bombus insularis* | 1 | 0 | | 0 | 1 | |
|  | Apidae | *Bombus rufocinctus* | 18 | 7 | | 8 | 33 | |
|  | Apidae | *Bombus suckleyi* | 0 | 1 | | 1 | 2 | |
|  | Apidae | *Bombus ternarius* | 11 | 11 | | 10 | 32 | |
|  | Apidae | *Bombus vagans* | 1 | 0 | | 0 | 1 | |
|  | Apidae | *Diadasia australis* | 10 | 15 | | 4 | 29 | |
|  | Apidae | *Diadasia diminuta* | 14 | 8 | | 4 | 26 | |
|  | Apidae | *Epeolus compactus* | 0 | 0 | | 1 | 1 | |
|  | Apidae | *Epeolus minimus* | 79 | 40 | | 21 | 140 | |
|  | Apidae | *Eucera fulvitarsis* | 4 | 2 | | 1 | 7 | |
|  | Apidae | *Eucera speciosa* | 4 | 3 | | 2 | 9 | |
|  | Apidae | *Melissodes agilis* | 17 | 12 | | 6 | 35 | |
|  | Apidae | *Melissodes confusus* | 6 | 5 | | 7 | 18 | |
|  | Apidae | *Melissodes* cf. *coreopsis* | 172 | 270 | | 135 | 577 | |
|  | Apidae | *Melissodes* cf. *druriella* | 1 | 2 | | 0 | 3 | |
|  | Apidae | *Melissodes microsticta* | 1 | 1 | | 0 | 2 | |
|  | Apidae | *Melissodes perlusa* | 2 | 3 | | 5 | 10 | |
|  | Apidae | *Melissodes rivalis* | 28 | 24 | | 36 | 88 | |
|  | Apidae | *Melissodes* cf. *semilupina* | 1 | 0 | | 0 | 1 | |
|  | Apidae | *Melissodes snowii* | 9 | 3 | | 0 | 12 | |
|  | Apidae | *Nomada articulata* | 1 | 0 | | 1 | 2 | |
|  | Apidae | *Nomada lehighensis* | 1 | 0 | | 0 | 1 | |
|  | Apidae | *Nomada* sp.1 | 0 | 0 | | 1 | 1 | |
|  | Apidae | *Nomada* sp.2 | 2 | 3 | | 1 | 6 | |
|  | Apidae | *Nomada* sp.3 | 0 | 1 | | 0 | 1 | |
|  | Apidae | *Nomada* sp.4 | 0 | 1 | | 0 | 1 | |
|  | Apidae | *Triepeolus balteatus* | 0 | 0 | | 1 | 1 | |
|  | Apidae | *Triepeolus helianthi* | 0 | 0 | | 1 | 1 | |
|  | Apidae | *Triepeolus micropygius* | 0 | 1 | | 0 | 1 | |
|  | Apidae | *Triepeolus* sp.1 | 0 | 2 | | 3 | 5 | |
|  | Colletidae | *Colletes* cf. *aberrans* | 1 | 0 | | 0 | 1 | |
|  | Colletidae | *Colletes brevicornis* | 25 | 32 | | 41 | 98 | |
|  | Colletidae | *Colletes hyalinus* | 51 | 33 | | 50 | 134 | |
|  | Colletidae | *Colletes impunctatus* | 10 | 23 | | 20 | 53 | |
|  | Colletidae | *Colletes kincaidii* | 13 | 9 | | 7 | 29 | |
|  | Colletidae | *Colletes nigrifrons* | 0 | 1 | | 6 | 7 | |
|  | Colletidae | *Colletes phaceliae* | 1 | 2 | | 2 | 5 | |
|  | Colletidae | *Colletes* cf. *simulans* | 0 | 9 | | 0 | 9 | |
|  | Colletidae | *Hylaeus affinis* | 0 | 0 | | 2 | 2 | |
|  | Colletidae | *Hylaeus annulatus* | 1 | 4 | | 0 | 5 | |
|  | Colletidae | *Hylaeus mesillae* | 24 | 12 | | 8 | 44 | |
|  | Colletidae | *Hylaeus modestus* | 1 | 0 | | 0 | 1 | |
|  | Halictidae | *Agapostemon femoratus* | 2 | 1 | | 0 | 3 | |
|  | Halictidae | *Agapostemon obliquus* | 0 | 1 | | 0 | 1 | |
|  | Halictidae | *Agapostemon splendens* | 1 | 2 | | 2 | 5 | |
|  | Halictidae | *Agapostemon texanus* | 72 | 55 | | 15 | 142 | |
|  | Halictidae | *Agapostemon virescens* | 100 | 90 | | 56 | 246 | |
|  | Halictidae | *Agapsotemon obliquus* | 1 | 0 | | 0 | 1 | |
|  | Halictidae | *Dufourea marginata* | 7 | 4 | | 11 | 22 | |
|  | Halictidae | *Dufourea maura* | 20 | 20 | | 59 | 99 | |
|  | Halictidae | *Halictus confusus* | 100 | 87 | | 33 | 220 | |
|  | Halictidae | *Halictus ligatus* | 1 | 5 | | 1 | 7 | |
|  | Halictidae | *Halictus rubicundus* | 71 | 62 | | 79 | 212 | |
|  | Halictidae | *Lasioglossum* cf. *albipenne* | 6 | 1 | | 2 | 9 | |
|  | Halictidae | *Lasioglossum anomalum* | 9 | 23 | | 7 | 39 | |
|  | Halictidae | *Lasioglossum egregium* | 1 | 1 | | 1 | 3 | |
|  | Halictidae | *Lasioglossum hudsoniellum* | 60 | 84 | | 13 | 157 | |
|  | Halictidae | *Lasioglossum* cf. *laevissimum* | 23 | 17 | | 12 | 52 | |
|  | Halictidae | *Lasioglossum leucozonium* | 22 | 21 | | 11 | 54 | |
|  | Halictidae | *Lasioglossum* cf. *nigroviride* | 12 | 19 | | 5 | 36 | |
|  | Halictidae | *Lasioglossum paraforbesii* | 97 | 47 | | 30 | 174 | |
|  | Halictidae | *Lasioglossum* cf. *pavoninum* | 15 | 12 | | 12 | 39 | |
|  | Halictidae | *Lasioglossum perpunctatum* | 71 | 60 | | 37 | 168 | |
|  | Halictidae | *Lasioglossum pruinosum* | 133 | 139 | | 102 | 374 | |
|  | Halictidae | *Lasioglossum* cf. *rufulipes* | 2 | 7 | | 1 | 10 | |
|  | Halictidae | *Lasioglossum sagax* | 102 | 94 | | 82 | 278 | |
|  | Halictidae | *Lasioglossum* sp.1 | 0 | 1 | | 0 | 1 | |
|  | Halictidae | *Lasioglossum* sp.2 | 0 | 0 | | 2 | 2 | |
|  | Halictidae | *Lasioglossum* sp.3 | 0 | 2 | | 0 | 2 | |
|  | Halictidae | *Lasioglossum succinipenne* | 362 | 435 | | 287 | 1084 | |
|  | Halictidae | *Lasioglossum testaceum* | 3 | 11 | | 2 | 16 | |
|  | Halictidae | *Lasioglossum zonulum* | 1 | 2 | | 0 | 3 | |
|  | Halictidae | *Sphecodes arroyanus* | 2 | 0 | | 0 | 2 | |
|  | Halictidae | *Sphecodes minor* | 2 | 2 | | 1 | 5 | |
|  | Megachilidae | *Anthidium clypeodentatum* | 10 | 9 | | 7 | 26 | |
|  | Megachilidae | *Anthidium tenuiflorae* | 9 | 6 | | 1 | 16 | |
|  | Megachilidae | *Coelioxys moestus* | 0 | 0 | | 1 | 1 | |
|  | Megachilidae | *Coelioxys rufitarsis* | 10 | 7 | | 27 | 34 | |
|  | Megachilidae | *Coelioxys sodalis* | 1 | 3 | | 2 | 6 | |
|  | Megachilidae | *Dianthidium pudicum* | 3 | 2 | | 0 | 5 | |
|  | Megachilidae | *Hoplitis pilosifrons* | 52 | 31 | | 25 | 108 | |
|  | Megachilidae | *Hoplitis producta* | 38 | 27 | | 6 | 71 | |
|  | Megachilidae | *Hoplitis robusta* | 0 | 0 | | 3 | 3 | |
|  | Megachilidae | *Hoplitis spoliata* | 1 | 6 | | 0 | 7 | |
|  | Megachilidae | *Megachile brevis* | 4 | 8 | | 19 | 31 | |
|  | Megachilidae | *Megachile centuncularis* | 1 | 0 | | 2 | 3 | |
|  | Megachilidae | *Megachile dentitarsus* | 6 | 11 | | 26 | 43 | |
|  | Megachilidae | *Megachile fortis* | 0 | 0 | | 1 | 1 | |
|  | Megachilidae | *Megachile frigida* | 0 | 0 | | 2 | 2 | |
|  | Megachilidae | *Megachile inermis* | 1 | 6 | | 0 | 7 | |
|  | Megachilidae | *Megachile latimanus* | 11 | 15 | | 12 | 38 | |
|  | Megachilidae | *Megachile manifesta* | 1 | 0 | | 0 | 1 | |
|  | Megachilidae | *Megachile melanophaea* | 17 | 9 | | 10 | 36 | |
|  | Megachilidae | *Megachile montivaga* | 3 | 3 | | 1 | 7 | |
|  | Megachilidae | *Megachile perihirta* | 5 | 5 | | 8 | 18 | |
|  | Megachilidae | *Megachile rotundata* | 39 | 31 | | 4 | 74 | |
|  | Megachilidae | *Megachile wheeleri* | 1 | 3 | | 0 | 4 | |
|  | Megachilidae | *Osmia* cf*. distincta* | 48 | 31 | | 26 | 105 | |
|  | Megachilidae | *Osmia integra* | 17 | 13 | | 6 | 36 | |
|  | Megachilidae | *Osmia* cf*. longula* | 2 | 2 | | 1 | 5 | |
|  | Megachilidae | *Osmia* cf*. proxima* | 0 | 1 | | 0 | 1 | |
|  | Megachilidae | *Osmia* cf*. simillima* | 7 | 19 | | 8 | 34 | |
|  | Megachilidae | *Osmia* sp.1 | 0 | 1 | | 2 | 3 | |
|  | Megachilidae | *Osmia* sp.2 | 19 | 13 | | 3 | 35 | |
|  | Megachilidae | *Osmia* sp.3 | 1 | 0 | | 2 | 3 | |
|  | Megachilidae | *Osmia* sp.4 | 6 | 2 | | 0 | 8 | |
|  | Megachilidae | *Osmia* sp.5 | 112 | 83 | | 56 | 251 | |
|  | Megachilidae | *Osmia* cf*. tersula* | 11 | 0 | | 0 | 11 | |
|  | Megachilidae | *Osmia texana* | 0 | 3 | | 1 | 4 | |
|  | Megachilidae | *Stelis lateralis* | 1 | 1 | | 1 | 3 | |
|  | Megachilidae | *Stelis nitida* | 0 | 0 | | 1 | 1 | |
|  | Megachilidae | *Stelis* sp.1 | 2 | 1 | | 0 | 3 | |
| *Species richness* | |  | *113* | *113* | | *106* | *147* | |
| *Abundance* | |  | *2524* | *2453* | | *1678* | *6645* | |
| **Papilionoidea** | | | | | | | | |
|  | Hesperiidae | *Anatrytone delaware* | 0 | 1 | | 1 | 2 | |
|  | Hesperiidae | *Anatrytone logan* | 10 | 7 | | 0 | 17 | |
|  | Hesperiidae | *Hesperia assiniboia* | 462 | 483 | | 796 | 1741 | |
|  | Hesperiidae | *Hesperia nevada* | 3 | 3 | | 0 | 6 | |
|  | Hesperiidae | *Oarisma garita* | 25 | 26 | | 30 | 81 | |
|  | Hesperiidae | *Ochlodes sylvanoides* | 7 | 10 | | 7 | 24 | |
|  | Hesperiidae | *Polites mystic* | 4 | 2 | | 0 | 6 | |
|  | Hesperiidae | *Polites peckius* | 2 | 6 | | 5 | 13 | |
|  | Hesperiidae | *Polites themistocles* | 1 | 2 | | 0 | 3 | |
|  | Lycaenidae | *Glaucopsyche lygdamus* | 0 | 4 | | 0 | 4 | |
|  | Lycaenidae | *Icaricia saepiolus* | 3 | 4 | | 3 | 10 | |
|  | Lycaenidae | *Lycaena helloides* | 0 | 0 | | 1 | 1 | |
|  | Lycaenidae | *Lycaena rubida* | 2 | 4 | | 1 | 7 | |
|  | Lycaenidae | *Lycaena rubidus* | 1 | 0 | | 0 | 1 | |
|  | Lycaenidae | *Plebejus melissa* | 23 | 17 | | 15 | 55 | |
|  | Nymphalidae | *Boloria bellona* | 1 | 0 | | 0 | 1 | |
|  | Nymphalidae | *Cercyonis pegala* | 20 | 13 | | 45 | 78 | |
|  | Nymphalidae | *Coenonympha california* | 32 | 48 | | 42 | 122 | |
|  | Nymphalidae | *Oeneis uhleri* | 13 | 6 | | 10 | 29 | |
|  | Nymphalidae | *Phyciodes tharos* | 1 | 1 | | 0 | 2 | |
|  | Nymphalidae | *Speyeria aphrodite* | 6 | 4 | | 8 | 18 | |
|  | Nymphalidae | *Speyeria callippe* | 1 | 2 | | 1 | 4 | |
|  | Nymphalidae | *Vanessa cardui* | 2 | 3 | | 3 | 8 | |
|  | Pieridae | *Colias alexandra* | 22 | 4 | | 0 | 26 | |
|  | Pieridae | *Colias philodice* | 78 | 60 | | 48 | 186 | |
|  | Pieridae | *Pieris rapae* | 14 | 28 | | 12 | 54 | |
|  | Pieridae | *Pontia occidentalis* | 2 | 8 | | 3 | 13 | |
|  | Pieridae | *Pontia protodice* | 2 | 0 | | 0 | 2 | |
| *Species richness* | |  | *25* | *24* | | *18* | *28* | |
| *Abundance* | |  | *737* | *746* | | *1031* | *2514* | |
| **Coleoptera** | | | | | | | | |
|  | Anthicidae | *Anthicus* sp. | 1 | 0 | | 0 | 1 | |
|  | Anthicidae | *Notoxus* cf. *anchora* | 100 | 171 | | 127 | 398 | |
|  | Cantharidae | *Cantharis* sp. | 2 | 0 | | 0 | 2 | |
|  | Carabidae | *Agonum ferruginosum* | 0 | 1 | | 0 | 1 | |
|  | Carabidae | *Amara littoralis* | 2 | 0 | | 0 | 2 | |
|  | Carabidae | *Amara sinuosa* | 0 | 1 | | 0 | 1 | |
|  | Carabidae | *Calosoma calidum* | 0 | 0 | | 1 | 1 | |
|  | Carabidae | *Cymindis cribricollis* | 0 | 0 | | 1 | 1 | |
|  | Carabidae | *Amara* (Curtonotus) sp. | 0 | 1 | | 0 | 1 | |
|  | Chrysomelidae | *Distigmoptera borealis* | 1 | 1 | | 0 | 2 | |
|  | Chrysomelidae | *Erynephala* cf. *puncticollis* | 1 | 1 | | 1 | 3 | |
|  | Chrysomelidae | *Pachybrachis hepaticus* | 4 | 0 | | 0 | 4 | |
|  | Chrysomelidae | *Phyllotreta* cf. sp. | 2 | 0 | | 0 | 2 | |
|  | Cleridae | *Phyllobaenus humeralis* | 7 | 9 | | 9 | 25 | |
|  | Cleridae | *Trichodes nuttalli* | 69 | 147 | | 222 | 438 | |
|  | Coccinellidae | *Brachiacantha albifrons* | 1 | 1 | | 1 | 3 | |
|  | Coccinellidae | *Hippodamia parenthesis* | 1 | 0 | | 2 | 3 | |
|  | Coccinellidae | *Hyperaspis inflexa* | 0 | 1 | | 0 | 1 | |
|  | Coccinellidae | *Hyperaspis lugubris* | 1 | 0 | | 0 | 1 | |
|  | Coccinellidae | *Hyperaspis undulata* | 0 | 0 | | 1 | 1 | |
|  | Coccinellidae | *Hyperaspis undulata* | 0 | 0 | | 1 | 1 | |
|  | Coccinellidae | *Scymnus* cf. *lacustris* | 1 | 0 | | 0 | 1 | |
|  | Curculionidae | *Acanthoscelidius* sp. | 11 | 11 | | 123 | 145 | |
|  | Curculionidae | *Baris* sp. | 1 | 1 | | 3 | 5 | |
|  | Curculionidae | *Cosmobaris scolopacea* | 1 | 0 | | 0 | 1 | |
|  | Curculionidae | *Glocianus punctiger* | 9 | 0 | | 0 | 9 | |
|  | Curculionidae | *Listronotus* sp. | 1 | 0 | | 0 | 1 | |
|  | Curculionidae | *Odontocorynus* sp. | 1 | 1 | | 0 | 2 | |
|  | Curculionidae | *Otiorhynchus ovatus* | 9 | 3 | | 3 | 15 | |
|  | Curculionidae | *Sciaphilus* cf. *asperatus* | 0 | 2 | | 1 | 3 | |
|  | Curculionidae | *Sitona cylindricollis* | 4 | 1 | | 1 | 6 | |
|  | Elateridae | *Aeolus mellillus* | 0 | 5 | | 0 | 5 | |
|  | Elateridae | *Hypnoidus* sp. | 2 | 0 | | 0 | 2 | |
|  | Elateridae | *Selatosomus aeripennis* | 2 | 0 | | 0 | 2 | |
|  | Histeridae | *Atholus falli* | 0 | 1 | | 0 | 1 | |
|  | Hydrophilidae | *Hydrobius fuscipes* | 1 | 0 | | 0 | 1 | |
|  | Meloidae | *Epicauta ferruginea* | 192 | 338 | | 85 | 615 | |
|  | Meloidae | *Epicauta pennsylvanica* | 1 | 2 | | 2 | 5 | |
|  | Meloidae | *Epicauta pruinosa* | 88 | 109 | | 115 | 312 | |
|  | Meloidae | *Epicauta puncticollis* | 10 | 7 | | 5 | 22 | |
|  | Meloidae | *Epicauta subglabra* | 1 | 0 | | 0 | 1 | |
|  | Meloidae | *Lytta nuttalli* | 0 | 1 | | 0 | 1 | |
|  | Meloidae | *Nemognatha lutea* | 1 | 1 | | 1 | 3 | |
|  | Melyridae | *Collops bipunctatus* | 1 | 1 | | 1 | 3 | |
|  | Melyridae | *Collops vittatus* | 5 | 2 | | 13 | 20 | |
|  | Melyridae | *Listrus* sp. | 2 | 0 | | 0 | 2 | |
|  | Miridae | *Hadronema* cf. *pictum* | 1 | 0 | | 0 | 1 | |
|  | Mordellidae | *Mordella atrata* | 10 | 20 | | 10 | 40 | |
|  | Mordellidae | *Mordellistena* sp.1 | 2 | 19 | | 4 | 25 | |
|  | Mordellidae | *Mordellistena* sp.2 | 7 | 2 | | 15 | 24 | |
|  | Mordellidae | *Mordellistena* sp.3 | 1 | 0 | | 0 | 1 | |
|  | Mordellidae | *Mordellistena* sp.4 | 5 | 10 | | 2 | 17 | |
|  | Mordellidae | *Mordellistena* sp.5 | 1 | 2 | | 1 | 4 | |
|  | Nitidulidae | *Nitops pallipennis* | 2 | 3 | | 7 | 12 | |
|  | Phalacridae | *Olibrus* sp. | 0 | 1 | | 0 | 1 | |
|  | Ptinidae | *Xyletinus* sp. | 1 | 0 | | 0 | 1 | |
|  | Pyrochroidae | *Pedilus* sp. | 0 | 1 | | 0 | 1 | |
|  | Scarabaeidae | *Cryptoscatomaseter* cf*. criddlei* | 0 | 1 | | 0 | 1 | |
|  | Scarabaeidae | *Dichelonyx truncata* | 1 | 1 | | 1 | 3 | |
|  | Scarabaeidae | *Diplotaxis obscura* | 0 | 0 | | 2 | 2 | |
|  | Scarabaeidae | *Flaviellus consentaneus* | 2 | 0 | | 2 | 4 | |
|  | Scarabaeidae | *Onthophagus nuchicornis* | 0 | 1 | | 0 | 1 | |
|  | Scarabaeidae | *Otophorus haemorrhoidalis* | 2 | 0 | | 0 | 2 | |
|  | Scarabaeidae | *Serica curvata* | 23 | 19 | | 19 | 61 | |
|  | Tenebrionidae | *Blapstinus metallicus* | 2 | 0 | | 0 | 2 | |
| *Species richness* | |  | 48 | 38 | | 32 | 65 | |
| *Abundance* |  |  | 596 | 900 | | 782 | 2278 | |
| **Total** |  |  |  |  | |  |  | |
|  | ***Species richness*** | | **186** | **175** | | **156** | **240** | |
|  | ***Abundance*** | | **3857** | **4099** | | **3491** | **11437** | |
