# Supplementary figures and images for: Biodiversity measures of a grassland plant-pollinator community are resilient to the introduction of honey bees (Apis mellifera)
Source: PLoS One. 2024 Oct 25;19(10):e0309939. doi: 10.1371/journal.pone.0309939 (PMC11508496; doi:10.1371/journal.pone.0309939)

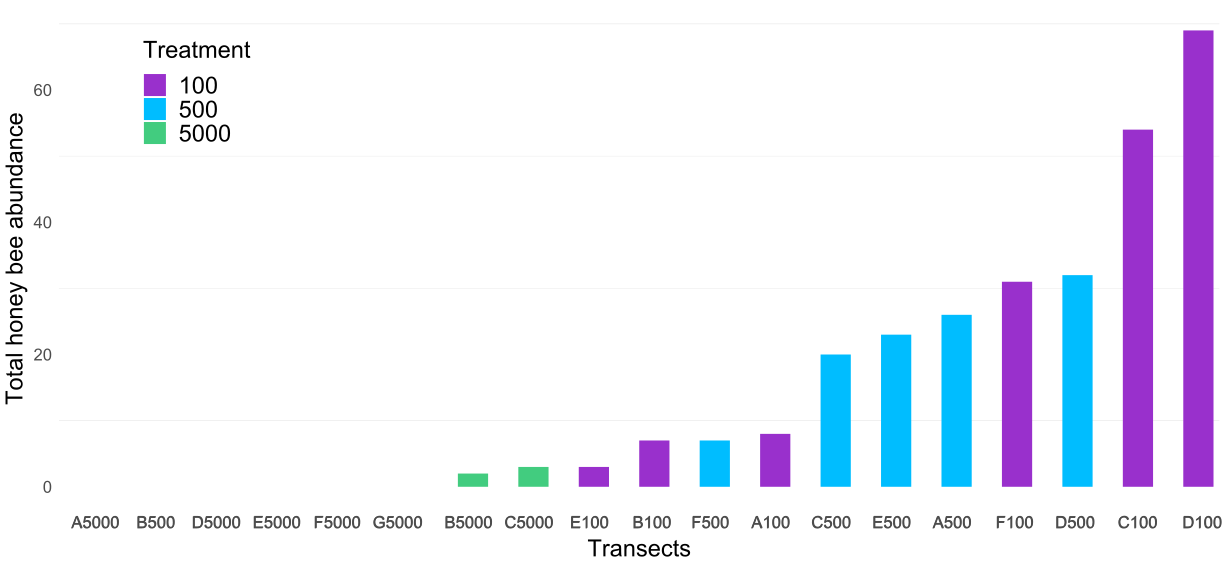

Supplement: S1 Fig — Honey bees were pooled across the full season per transect, with transects ordered by increasing honey bee abundance, and coloured by distance from bee hives. In the transect names, 100 indicates 100 m, 500 indicates 500 m, and 5000 indicates 5000 m distances from hives. The Eastern Irrigation District, owner of the land surrounding the Mattheis Research Ranch, reported no known apiaries within 19 km of the ranch’s boundary line. All commercial or hobbyist apiaries are required by law to register their hive(s) annually to the Provincial Apiculturist. Reprinted from Worthy et al. [29] under a CC BY license, with permission from PLOS ONE, original copyright 2023. (TIF) [file pone.0309939.s009.tif]

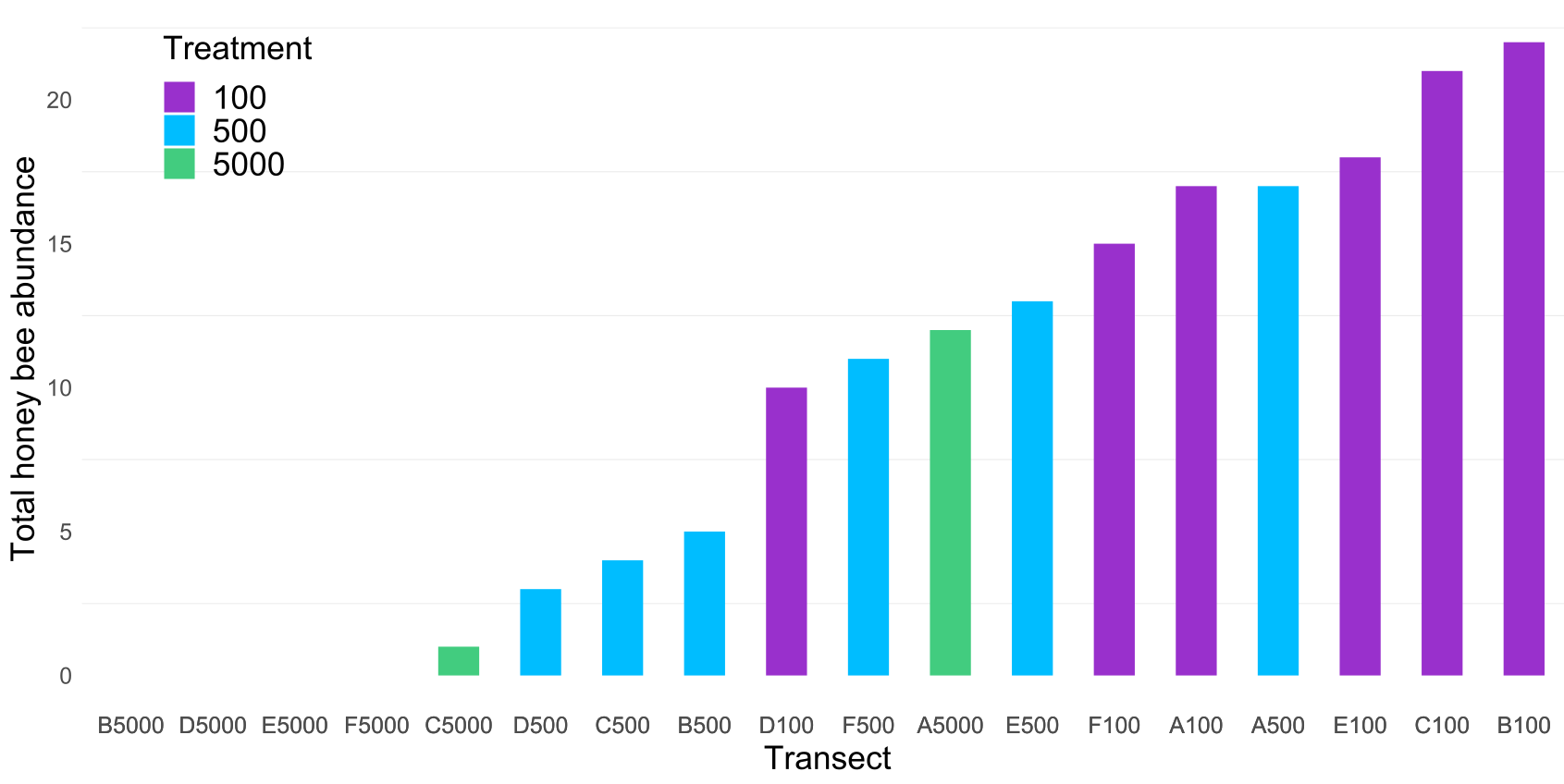

Supplement: S2 Fig — In the transect names, 100 indicates 100 m, 500 indicates 500 m, and 5000 indicates 5000 m distances from hives. Reprinted from Worthy et al. [29] under a CC BY license, with permission from PLOS ONE, original copyright 2023. (TIF) [file pone.0309939.s010.tif]

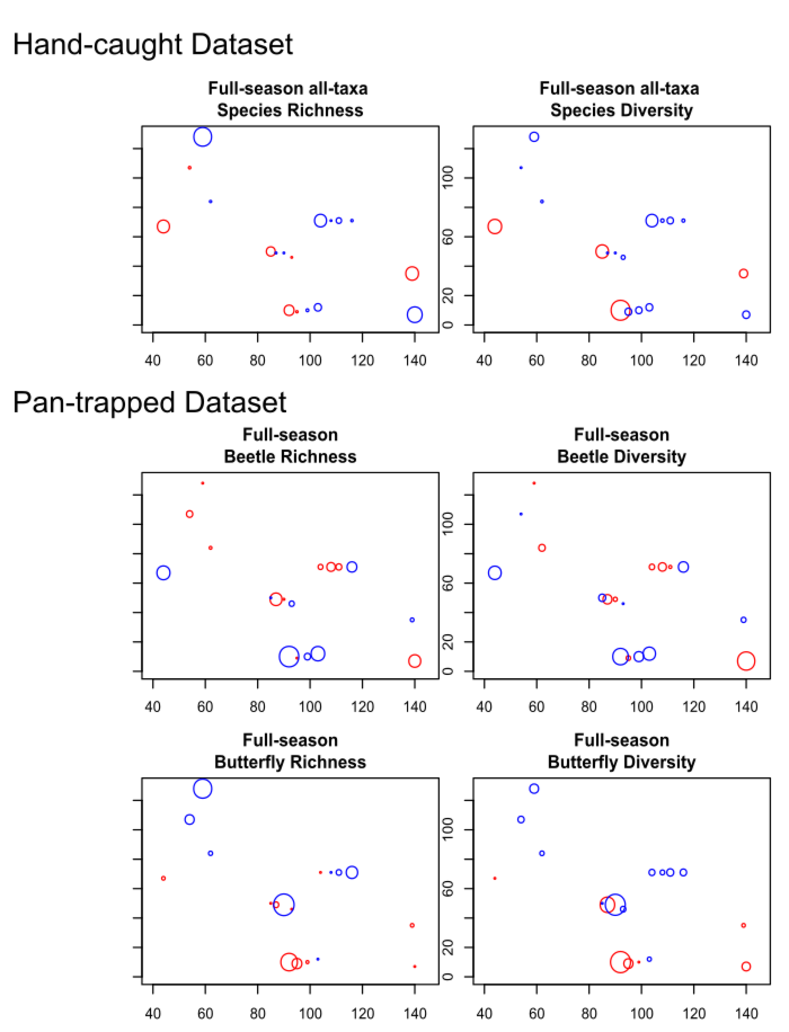

Supplement: S3 Fig — Circles indicate the size of the residual for each transect (smaller circles = better model fit). Colour indicates the sign of the residual; blue shows values lower than 0 and red values higher than 0. In this figure, if close together transects have similarly sized and coloured residuals, that suggests that there is spatial autocorrelation in that response variable. Reprinted from Worthy et al. [29] under a CC BY license, with permission from PLOS ONE, original copyright 2023. (TIF) [file pone.0309939.s011.tif]

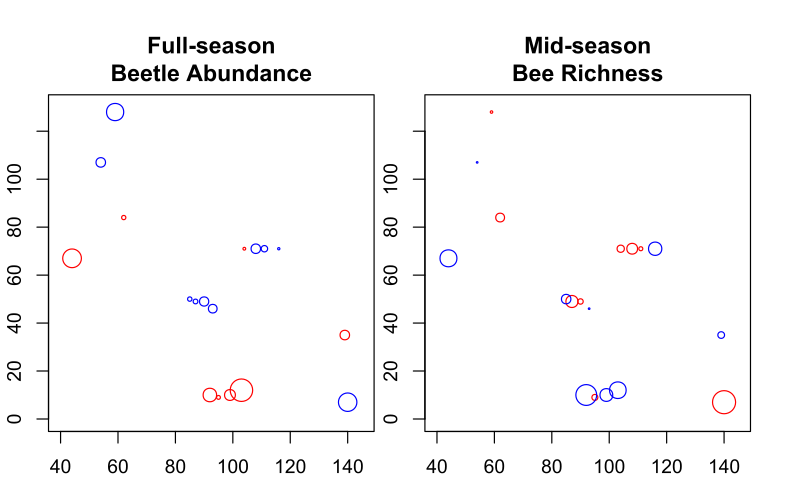

Supplement: S4 Fig — Both models required rational quadratic special correlation structures. Circles indicate the size of the residual for each transect in the model without any special correlation structure (smaller circles = better model fit). Colour indicates the sign of the residual; blue shows values lower than 0 and red values higher than 0. In this figure, if close together transects have similarly sized and coloured residuals, that suggests that there is spatial autocorrelation in that response variable. Reprinted from Worthy et al. [29] under a CC BY license, with permission from PLOS ONE, original copyright 2023. (TIF) [file pone.0309939.s012.tif]

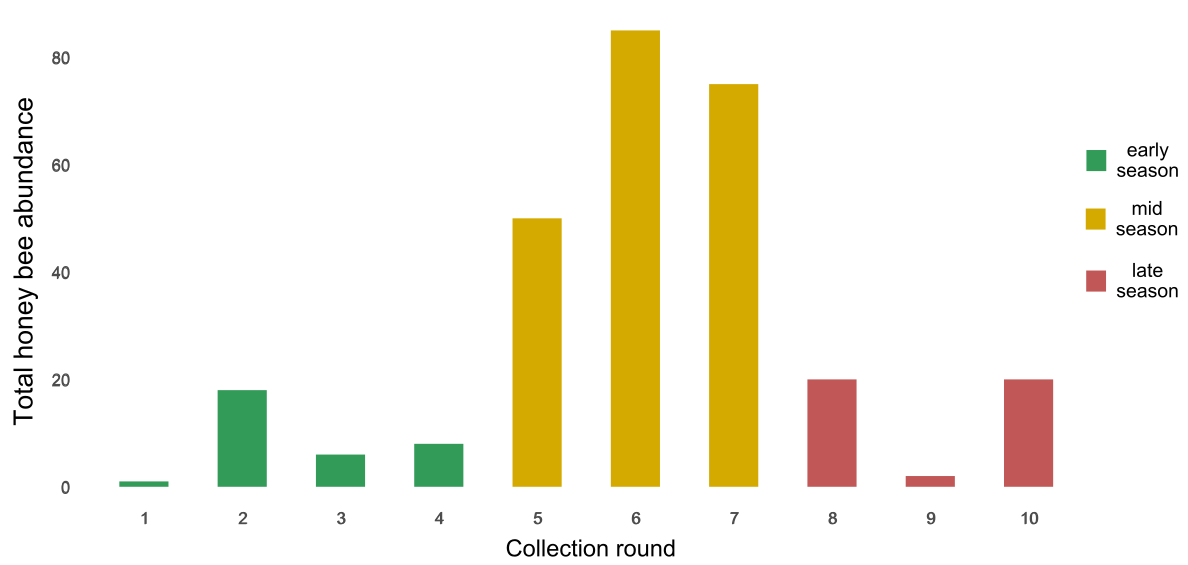

Supplement: S5 Fig — The entire season’s collection rounds were split into three groups: collection rounds 1–4 represented “early” season (May 28th-July 7th), 5–7 represented “mid” season (July 8th-July 31st), and 8–10 represented “late” season (August 1st-August 28th). Reprinted from Worthy et al [29] under a CC BY license, with permission from PLOS ONE, original copyright 2023. (TIF) [file pone.0309939.s013.tif]

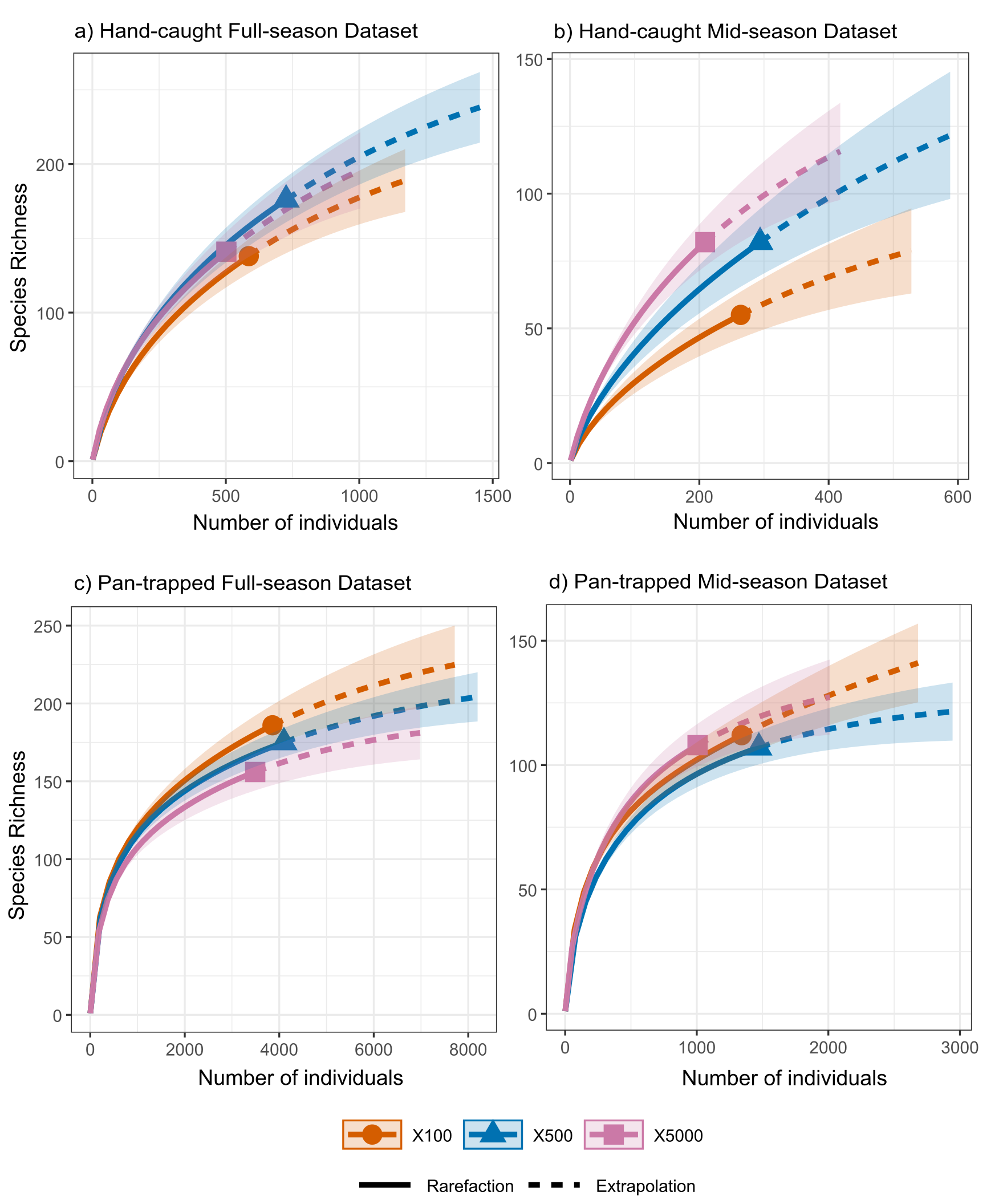

Supplement: S6 Fig — Species rarefaction curves for a) full-season all-taxa dataset (1), b) mid-season all-taxa dataset (4), c) pan-trapped higher taxa dataset (6,7, and 8), and d) mid-season pan-trapped higher taxa dataset (9 and 10), where the richness of each higher taxon in the pan-trapped dataset (bees, beetles, butterflies) were pooled together. These curves plot the average number of species obtained from repeated random re-sampling of the number of individuals given on the x-axis. Transects were pooled by distance from hives, as indicated by green (100 m), red (500 m), and blue (5000 m). Confidence intervals indicate 95%. Figures were generated with the iNEXT package. (TIF) [file pone.0309939.s014.tif]
