# Supplementary material for: Biodiversity measures of a grassland plant-pollinator community are resilient to the introduction of honey bees (Apis mellifera)
Source: PLoS One. 2024 Oct 25;19(10):e0309939. doi: 10.1371/journal.pone.0309939 (PMC11508496; doi:10.1371/journal.pone.0309939)
Supplement: Attachment — Submitted filename: Response to Reviewers.docx [file pone.0309939.s015.docx]

Dear Dr. Iqbal,

Thank you for allowing us the opportunity to re-submit our manuscript to *PLOS ONE*. We have revised our manuscript “Biodiversity measures of a grassland plant-pollinator community are resilient to the introduction of honey bees (*Apis mellifera*)” in light of the reviewers’ comments, and we believe that the manuscript is improved as a result. We are grateful for these comments and have responded specifically to each suggestion. We have marked our responses with ‘>’. Additionally, we have addressed the journal requirements listed in the decision letter and added a full ethics statement in the Methods section, as well as have addressed the copyright issues regarding the figures and tables.

We thank you for your time and consideration.

Yours sincerely,

Sydney Worthy

John Acorn

Carol Frost

Reviewer #1 (Comments to Author): 

The article entitled "Biodiversity measures of a grassland plant-pollinator community are resilient to the introduction of honey bees (Apis mellifera)" evaluates the effect of honey bee (Apis mellifera) density on diversity, abundance, species composition of wild pollinators and on the diversity of plant-wild pollinator interactions in the Canadian prairies. Due to habitat loss, the negative effects of pesticides, the increase in bee diseases, etc., wild pollinators are at serious risk of declining populations and diversity, so studies to better understand the effects and risks to wild pollinators can contribute to their conservation at a global level.

These risks are also suffered by honey bees, which are managed by beekeepers who are forced to introduce them into natural areas in search of pollen resources for food and production. The increase of honey bee hives in natural areas and their effects on wild pollinators is a topic of great importance at present, about which many factors are still unknown, so studies such as the present work are necessary to be able to make management plans on the introduction of honey bee hives in natural areas.

The article is well written and organised. The introduction sets out the background to the work, the objectives in an appropriate manner. The methodology used is well explained and detailed.

**> We thank the reviewer for these positive comments.**

In general, the objectives have been completed correctly, with an adequate methodology and with a correct exposition of the results. However, the fourth objective (determine whether effects of honey bees are greater when honey bee colony sizes are larger, and whether this differs for different higher taxa), is the one that could be methodologically improved by including some more data. To know more exactly the density of honey bees in the territory, the census of hives in the vicinity should be known and taken into account.

**>We thank the reviewer for this idea and agree it would be ideal to have greater certainty on the exact number of hives in the region of the Mattheis Research Ranch. However, we did obtain and present this information in as much detail as possible. We have now made this information more prominent, however, by moving it to the methods. We now report on L126-128, “the Eastern Irrigation District, owner of the land surrounding the Mattheis Research Ranch, reported no known apiaries within 19 km of the ranch’s boundary line within the study year or the previous year”.**

**There was also a natural “barrier” of the Red Deer River, which borders the northern and eastern edges of the Ranch and has a tributary on the southern edge. The river is approximately 225 m wide on average and while we cannot be sure of the extent it deters insect travel, there is evidence that rivers discourage flying insects (Sciarretta & Trematerra 2006, Journal of Applied Entomology, Volume 130, Issue 2, Pages 73-83) even if not insurmountable (Zurbuchen et al., 2010, Journal of Animal Ecology, Volume 79, Issue 3, Pages 674-681).**

**Additionally, because we drove to 20 km from the Mattheis Research Ranch in all driveable directions and did not observe any apiaries from the road,** **we feel that we confirmed adequately (and as well as it was possible to do) that large-scale commercialized honey bee operations were not close enough to the study site to invalidate our design. Feral hives, and illegal, unregistered hives, may have been possible, as we mention on L193.**

**However, information on the exact density of honey bee colonies in the region would be more important to the validity of our study if we had used distances from hives as the predictor variable representing honey bee density. However, we did not do this, partly because we did not have perfect certainty as to whether feral or unregistered hives could have been present on the landscape.** **Rather, we used the number of hand-caught honey bees on our transects. Thus, we used an empirically-measured estimate of honey bee density at the transects where we were measuring wild pollinator abundance, diversity and interactions. This is because honey bee foragers share information about resource location and quality, such that we would not expect distance alone to explain honey bee activity around flowers, but also floral patch quality. As such, we believe that our choice of predictor variable negates this concern, especially as it allowed us to realize that one of our originally selected 5000-m sites was unexpectedly receiving many visits from honey bees, and to relocate it to another location, where there was negligible honey bee activity. If unknown, additional honey bee colonies had contributed interactions to our transects, we should have detected them during sampling as we did at the transect F5000.**

As well as the state of development of the honey bee hives introduced in the study, i.e., data on the increase of brood frames, percentage of workers per frame, weight of the hives, production at the end of the season, etc., should be included in order to have objective indexes of the growth of the hive and be sure that this leads to an increase in the density of honey bees throughout the season, since any sanitary, nutritional or management problem can produce a population decrease and the death of the hive. It is recommended that such data be included in the analysis.

**>We agree with the reviewer that this information would support the data; however, it must be considered that this information is now impossible to retrieve. During the study, we confirmed bi-weekly, with the apiarist, that the hives were healthy at the study sites. Additionally, we visited the hives weekly to ensure that no animals had disturbed them, that swarms were not starting, and that the hives appeared active. We have now added: “The hives were assessed bi-weekly by the apiarist for health and productivity throughout the season. By late summer, all hives grew significantly and had 4 to 6 supers added per hive.”** **(L120-122).**

A question for the authors:

What is the average size of an apiary in Canada, as professional apiaries between 16-48 hives seem to me to be of a small size, so the effects of large apiaries (larger than 150 hives) have not been studied in the present work and should be mentioned in the discussion and conclusion. The present work is valid only for small size apiaries.

**>We agree with the reviewer that this would be a small-scale operation and thus have adjusted the language to suit this change (L120).**

Other considerations:

In Figure 2, there is an error in the name: (8) Full-season Butterflies.

**>We believe this mark may have appeared while converting the image file in the PLOS ONE PACE image conversion services. We will ensure it is not present upon resubmission.**

- In figure 3, indicate in the graph the regression line data, confidence intervals, etc...

**>We have adjusted Figure 3 to include regression line data and confidence intervals, as suggested.**

Reviewer #2 (Comments to Author):

In this manuscript Worthy et al. aim to investigate the effects of honey bees densities on a wild pollinator community of a Canadian grassland. Although the dataset is interesting, the paper does not properly justify why this new exploration of the data is needed considering the already published paper "Honey bees (Apis mellifera) modify plant-pollinator network structure, but do not alter wild species’ interactions" by the same authors. In lines 83-87 the authors write a few lines in this regard but these are not enough to justify a new publication.

**> We appreciate this concern, but the current manuscript reports a completely different analysis of the same data as Worthy et al. 2023, and also adds an entirely new dataset: the pan-trapped dataset. Worthy et al. 2023 modelled the plant-pollinator interactions measured as described here as a network (which we do not do here), and then tested whether the structural metrics that described this network changed as honey bee abundance increased. However, reviewers of that manuscript commented that it would be possible for pollinators to disappear or change in relative abundance (i.e., pollinator richness or diversity could decrease), or for certain interactions to disappear (i.e. interaction richness and diversity could decrease) even without significant changes to the network metrics reported. This is true, as network metrics are not fully dependent on biodiversity metrics. (For a striking example of this, see Tylianakis et al. 2007. Nature 445: 202-205.) As such, the biodiversity results reported here are a separate and equally important part of the story that was not reported in Worthy et al., 2023, simply because that paper already included a huge number of models, and it was not possible to include everything in one reasonably-lengthed paper.**

**To explain it another way, Worthy et al., 2023 reports effects of honey bee abundance on functional characteristics of the entire pollinator community, whereas the current manuscript reports on effects of honey bee abundance on biodiversity of the wild pollinator community.**

**We agree strongly with Reviewer 3 that publication of these “negative or null” results on this topic are very important, because there are so many studies reporting negative effects honey bees on exactly the metrics reported in the current manuscript. It is not possible to tell from reading Worthy et al., 2023 that the wild pollinator diversity and interaction diversity (altogether or for higher taxa separately) did not change as honey bee abundance increased, which is what we report here.**

**This said, the reviewer’s comment is useful, because it is important that the distinction between the two studies is clear, and we have improved our justification of the need for this further analysis of some of the data used in Worthy et al., 2023 (L112-116).**

The methods have a high overlap with the previously cited publication, including several identical paragraphs. On top of this there are a number of concerns and unclarities in the methods and statistics. Because of the above I consider that in its current form the manuscript is unsuitable for publication.

**>We have now modified the methods section of our paper to address the concern of high overlap. The reviewer did not explain what they found unclear, but we have edited the entire section for clarity.**

Reviewer #3:

The authors present a comprehensive study on the impact of introduced honeybees on the plant-pollinator community by analyzing several diversity metrics on different wild pollinators. The result obtained adds an interesting point of view and feeds the scientific debate on the effect of honeybees. I think it is important to publish this type of articles with negative or null results for scientific progress. However, in the present form, the manuscript has some shortcomings that prevent its publication.

**> We thank the reviewer for these positive comments and agree that negative/null results are important.**

The main objection is about the methodological approach used. Since the abundance and richness of floral resources is probably playing a relevant role as a predictor variable of pollinator diversity metrics, I do not know if the most appropriate methodological approach is to start analyzing the effect of honeybee abundance on SLRs and then explore with MR, the effect of other variables as predictor variable (with floral resources among them). Given honeybees' capacity to exploit high-abundance floral resources because of their ability to recruit nest-mates, both variables could be correlated. I believe that a more proper approach would be to first analyze the correlation between the different variables and then explore with MR different predictor variables avoiding the correlated ones (using the VIF).

**> We respectfully disagree, which we explain below, but we have still calculated VIF for interest (L281-285), and have added them to the supplement to show that they are not high enough to affect our results or interpretations.**

**We agree with the reviewer that in some applications of multiple regression, VIF are useful, because they show the degree to which each predictor variable is linearly correlated with the full set of other predictor variables in the model. If the VIF for a given predictor variable is high, the variance associated with that predictor variable in the model analysis is high, and therefore the P-value has substantially less likelihood of being significant in the multiple regression (MR) than if that predictor variable had been the only predictor variable in a simple linear regression (SLR) with the same response variable.**

**VIF are useful for knowing if the other covariates in the model are masking the effect of a focal predictor variable on the response variable. We have fully addressed this issue using another approach, which we believe is superior, because it doesn’t involve leaving out any predictor variables that we want to control for, which is what the reviewer is suggesting, should one or more of these variables create VIF that are “too high”. While we acknowledge that some statistics textbooks advocate the blanket application of VIF to multiple regression analysis, the VIF literature itself advocates more thoughtful use of VIF, including the statement they are not always useful, depending on the purpose of the analysis (e.g., O’Brien, R.M. 2007. A caution regarding rules of thumb for variance inflation factors. Quality & Quantity 41: 673-690), which some statistics textbooks also state (e.g., Jones, Hardin, & Crawley. 2023. “The R Book”. Third Edition. John Wiley & Sons Ltd. NJ, USA).**

**The problem that the reviewer mentions of potential multicollinearity between our predictor variable of interest (honey bee abundance) and the predictor variables that we included in the MR models as controls (flower abundance, flower species richness, and collection effort), is exactly the reason why we used the two-step approach of first running an SLR testing the effect of honey bee abundance alone on each response variable (not controlling for other potentially correlated variables that might also affect the response variable), and then running an MR model that includes all these other variables. The SLR results show the maximum possible effect size of honey bee abundance on each response variable (Morrissey & Ruxton, 2018). These SLR models are therefore effectively the least conservative assessment of the effect of honey bee abundance on each response variable. They are less conservative than the reviewer’s suggestion of using VIF to select some additional variables to put in the model. We wish to include this least conservative view of the potential effect of honey bee abundance on each response variable, because we believe it is important. This is because, in MR models, it is impossible to distinguish where the true causation lies when multiple predictor variables are correlated but significantly related to the response variable (Morrissey & Ruxton, 2018). As such, if we used VIF to exclude variables that had high collinearity with one or more other variables, we could be excluding the causative variable and keeping the non-causative but correlated variable.**

**Because of the inability of multiple regression to distinguish correlation and causation, we believe that the best we can do at assessing effects of honey bee abundance on each response variable is to look at the maximum possible effect (its regression coefficient in the SLR; least conservative) and its effect after controlling for every other potentially correlated variable (its partial regression coefficient in the MR; most conservative). The true effect of honey bee abundance on the response variable should lie somewhere between these two coefficient values.**

**We would also like to point out that in no case did the sign of the coefficient for honey bee abundance change in going from the SLR to the MR for a given response variable. Thus, we were not getting “odd” results for the effect of honey bee abundance from our MR models, which is sometimes a reason to use VIF to reduce variables in an MR model.**

**The two sets of relevant VIFs for our models are those calculated from the full-season data and those calculated from the mid-season data (which we now present as Table S4 in the supplement):**

| **Full Season** | |
| --- | --- |
| **Predictor variable** | **VIF** |
| Honey bee abundance | 3.59 |
| Flower species richness | 1.96 |
| Flower abundance | 3.51 |
| Collection effort | 1.41 |
| **Midseason** | |
| **Predictor variable** | **VIF** |
| Honey bee abundance | 5.54 |
| Flower species richness | 1.82 |
| Flower abundance | 5.48 |
| Collection effort | 1.24 |

**The highest VIF in this table (5.54, for honey bee abundance in the midseason models), which means that the other three covariates explain 81.95% of the variation in honey bee abundance for the midseason analyses. This does not compromise the results of our analysis (and would not even if the VIF was much higher), because we have used an approach that accounts for this (as explained above). What it does mean is that we can’t be exactly sure of the size of the effect of honey bee abundance in midseason (which was already acknowledged). However, as all of our coefficients for the effect of honey bees were positive, whether in SLRs or MRs, we can say that we found no evidence of negative effects of honey bee abundance on any response variable.**

I have listed some comments and suggestion below.

Introduction

Line 46: “where they are not native”. Also, hb are widely used within its native range. This sentence may lead the reader to interpret impacts occur only in areas where it has been introduced and not in its native range.

**>We have adjusted this sentence to be clear that honey production and crop pollination occurs in regions where honey bees are also native (L46).**

Line 48-50: rephrase.

**>We have altered L48-50 to be more clear to the reader.**

Materials and Methods
The three clusters are 3 km apart, which is closer than the maximum distance for each cluster (5km). Then make it clear the distances among 5000m hives, and how you deal with this effect. For example, E5000 is closer to A500 or A100 than to E500 or E100.

**>Since we didn’t use hive clusters and their associated transects of the same letter as statistical blocks, but rather used model selection on different correlation structures to account for potential spatial non-independence between transects, this concern is already accounted for in our statistical analysis. Note also that there were not hives at the 5000-m distances; there were only sampling transects. The only locations with hives were the white diamonds in Figure 1, as shown in the legend.**

As stated in the discussion maybe is better to describe in this section that there were no known apiaries within 19 km of the sampling area, that could have an effect on the experimental design (mainly on 5000m sites).

**>We have added the comment about honey bees not being within 19 km of the boundary line to the Methods section (L126-128).**

Why haven’t you analyzed the full dataset using both hand-caught and pan-trapped pollinators? The same in pan-trapped dataset: why do not use a full-season and mid-season with all-taxa dataset?

**>While we agree it would have been ideal to identify all pollinator groups within the pan-trapped material, there were so many specimens from the pan traps that we had to reduce it to specific groups: beetles, butterflies, and bees. In the mid-season, there were not enough flies in both the hand-caught and pan-trapped dataset, and with butterflies in the pan-trapped dataset, to be used in analyses.**

Results
I believe that figure 2 can be eliminated as it is not providing relevant information and can be replaced by another figure that provides an understanding of the results.

**> We respectfully disagree, because when there are so many datasets, it can be beneficial for a reader to go back and quickly visualize which dataset is being discussed. We request to keep it. However, if the editor wishes, we will move Figure 2 to the supplementary information or, if necessary, it can be completely removed.**

I have missed in the section "Effect of hb on pollinator diversity metrics" a first descriptive part detailing the richness and abundance of the different groups in the 3 distance categories (100,500 and 5000). Perhaps to avoid a greater number of tables, it can be included in table S3 or S5,or S7 summarized by group/family, so that the reader can check the raw data.

**> We have added species richness and abundance in both Tables S6 and S7, for higher taxa separately and in total. However, we do not wish to add this description to the main text, as we believe it will cause confusion about what was analyzed. This is because, as explained in the methods, we did not use distance from hives as the predictor variable representing honey bee density, but instead we used honey bee abundance in the hand-caught insects at each transect as the predictor variable representing honey bee density.**

In the fig S6 it seems that in the Mid-season dataset there seem to be an effect on pollinator richness hand-caught between the three distances. Have you explored this?

**>We thank the reviewer for this suggestion. In response to this comment we have added confidence intervals to Figure S6. We have added the following description of this trend on L350-354: “For all datasets, the 95% confidence intervals for the rarefaction curves overlapped for all distances from hives, though overlap was lowest for the mid-season hand-caught dataset, especially for the 100-m and 500-m distances (Fig. S6b). Only in this dataset was there a trend toward pollinator richness being highest at the 5000 m distance and lowest at the 100-m distance.”**

**We also refer to this in the discussion on L438.**
