# Supplementary material for: Biodiversity measures of a grassland plant-pollinator community are resilient to the introduction of honey bees (Apis mellifera)
Source: PLoS One. 2024 Oct 25;19(10):e0309939. doi: 10.1371/journal.pone.0309939 (PMC11508496; doi:10.1371/journal.pone.0309939)
Supplement: Attachment — Submitted filename: Response to Reviewers.docx [file pone.0309939.s016.docx]

Dear Dr. Iqbal,

Thank you for allowing us the opportunity to re-submit our manuscript to *PLOS ONE*. We have revised our manuscript “Biodiversity measures of a grassland plant-pollinator community are resilient to the introduction of honey bees (*Apis mellifera*)” in light of the reviewers’ comments. We are grateful for these comments and have responded specifically to each suggestion. We have marked our responses with ‘>’.

We thank you for your time and consideration.

Yours sincerely,

Sydney Worthy

John Acorn

Carol Frost

**Reviewer #1:** The authors have improved the manuscript and included the reviewers' corrections, so that it is now suitable for publication.

**> We thank the reviewer for this positive review.**

**Reviewer #4:** Minor linguistic revision.

**> Since no specific section needing revision was mentioned, we reread the whole manuscript focusing on linguistic clarity. In the end, we revised only the point mentioned below.**

**Reviewer #5:** Two minor corrections:
1- Adding keywords to the MS
2- Table 2 caption the word contained is repeated twice.

**>We have modified the keywords in editorial manager to better reflect the language used in the manuscript by changing the keyword “mixedgrass prairie” to “prairie”.**

**>We have removed the repeated word “contained” from the caption of Table 2 (L377).**
